# Survival of Lung Cancer Patients Dependent on the LOH Status for *DMP1*, *ARF*, and *p53*

**DOI:** 10.3390/ijms21217971

**Published:** 2020-10-27

**Authors:** Elizabeth A. Fry, Gloria E. Niehans, Robert A. Kratzke, Fumitake Kai, Kazushi Inoue

**Affiliations:** 1 Dept. of Pathology, Wake Forest University School of Medicine, Medical Center Blvd., Winston-Salem, NC 27157, USA; eafry10@hotmail.com (E.A.F.); fkai@wakehealth.edu (F.K.); 2 Minneapolis VA Medical Center, Minneapolis, MN 55417, USA; nieha002@umn.edu; 3 Dept. of Medicine, University of Minnesota Medical Center, Masonic Cancer Institute, Minneapolis, MN 55455, USA; kratz003@umn.edu

**Keywords:** DMTF1, *ARF*, INK4a, p53, Cyclin D1, YY1

## Abstract

Lung cancer is the leading cause of cancer deaths in the world, and accounts for more solid tumor deaths than any other carcinomas. The prognostic values of *DMP1*, *ARF*, and p53-loss are unknown in lung cancer. We have conducted survival analyses of non-small cell lung cancer (NSCLC) patients from the University of Minnesota VA hospital and those from the Wake Forest University Hospital. Loss of Heterozygosity (LOH) for h*DMP1* was found in 26 of 70 cases (37.1%), that of the *ARF/INK4a* locus was found in 33 of 70 (47.1%), and that of the *p53* locus in 43 cases (61.4%) in the University of Minnesota samples. LOH for h*DMP1* was associated with favorable prognosis while that of *p53* predicted worse prognosis. The survival was much shorter for *ARF*-loss than *INK4a*-loss, emphasizing the importance of *ARF* in human NSCLC. The adverse effect of *p53* LOH on NSCLC patients’ survival was neutralized by simultaneous loss of the h*DMP1* locus in NSCLC and breast cancer, suggesting the possible therapy of epithelial cancers with metastatic ability.

## 1. Introduction

Lung cancer is the leading cause of cancer deaths in the world, and accounts for more solid tumor deaths than any other carcinomas. More than 22,800 new cases are diagnosed each year in the United States alone, of which 135,700 will die 2020, representing 25% of all cancer deaths [1]. Lung cancer can be categorized into two major histopathological groups: Non small-cell lung cancer (NSCLC) and small-cell lung cancer (SCLC) [2], the latter of which shows neuroendocrine features. A total of 80%–85% of lung cancers are NSCLC [2], and they are subcategorized into adenocarcinomas (AC), squamous cell carcinomas (SCC), adenosquamous carcinomas, and large-cell carcinomas [3]. SCLC and NSCLC show major differences in histopathologic characteristics that can be explained by the distinct patterns of genetic alterations found in both tumor classes [4].

The *ARF* gene was cloned as the alternate reading frame gene for *p16^Ink4a^* from the mouse *ARF/Ink4a* locus [5,6,7,8,9,10]. Accumulating studies showed that the *ARF* tumor suppressor is a sensor for hyperproliferative oncogenic stimuli stemming from mutant Ras, c-Myc, E2F-1, and HER2 proteins [11,12,13,14], virtually induced by all the oncogenes stimulating p53 to induce cell cycle arrest, apoptosis, and autophagy [15]. p19*^ARF^* (p14*^ARF^* in humans) and p16^Ink4a^ mRNAs are generated from separate and first exons 1β and 1α (19.4 kilo base pairs (kbp) apart in humans; 12.4 kbp apart in mice) which splice into two common exons 2 and 3 [5]. These two genes are different since *p19^ARF^* uses only exons 1 and 2 (also p14*^ARF^*) while *p16^INK4a^* uses all of the exons 1–3 for production of the protein [5,16]. This *ARF-INK4a* (*CDKN2a*) locus is located 11.5 kbp downstream of the genomic locus for *CDKN2b* that encodes for p15^INK4^ [9]. All of *p15^Ink4b^*, *p19^ARF^*, and *p16^Ink4a^* are tumor suppressor genes as proved by analyses of gene knockout mice [17,18,19,20]. Since RB is regulated by p16^INK4a^ and p53 is regulated by p14*^ARF^*, the *ARF/INK4a* locus is very frequently inactivated in human cancers, second only to p53 [21,22]. *ARF* is a highly basic, insoluble protein (pI 11; [23]). Although human and mouse *ARF* differ in size (mouse 19 kDa, human 14 kDa) and show only 49% identity in amino acid sequences, the functions of the *ARF* proteins are well-conserved between the two species [6]. *ARF* is regulated at both transcriptional and protein levels by ULF, MKRN1, and SIVA1 [10].

Ectopic *ARF* arrests immortal rodent cell lines, such as NIH 3T3, as well as human cells from cancer [5,16,24]. The ability of *ARF* to inhibit cell cycle progression in a number of cell types suggested that *ARF* has powerful growth-inhibitory functions in cells, which stimulated researchers to study the in vivo activity of *ARF* to prevent tumors. *ARF* sequesters MDM2 in the nucleolus, thus preventing p53 degradation [25]. In addition, it inhibits the transcription factor E2F activity [26]. These activities lead to cell cycle arrest at G1 and G2 [16]. Itahana et al. studied the role of *ARF* in apoptosis and found that the mitochondrial protein p32/C1QBP bound to the *ARF* C-terminus, where p32 is required for *ARF* to localize to mitochondria to induce apoptosis, demonstrating the essential role of *ARF* in tumor suppression and programmed cell death [27]. Recent studies indicate that nuclear factor E2-related factor 2 is a major target of *ARF* in p53-independent tumor suppression [28].

The gene for the Dmp1 (cyclin D binding myb-like protein 1; Dmtf1) transcription factor was isolated in yeast two-hybrid screen of CTLL2 cell library with cyclin D2 bait [29]. Inoue and Sherr reported that gene expression and cell cycle arrest mediated by Dmp1 (Dmp1α) was antagonized by D-type cyclins through a Cdk-independent mechanism [30]. Importantly, Dmp1 directly binds to the Ets consensus sequence 5′ CCCGGATGC-3′ of the *ARF* promoter to activate its gene expression, thereby inducing p53-dependent cell cycle arrest ([29,30,31,32,33,34,35,36,37,38,39,40,41,42,43,44,45,46,47] for primary articles; [48,49,50,51] for reviews). The human *DMP1* locus encodes *DMP1α*, and its splice variants *DMP1β* and *γ* [46]. DMP1α regulates the human *ARF* promoter, the activity of which is antagonized its splice variant DMP1β [46]. Similar mechanisms must be present in human *ARF* promoter since both E2F and DMP1-consensus sequences are found in the human version as well. *Dmp1*-deficient mice were prone to spontaneous tumor development, which was accelerated when the animals were neonatally treated with ionizing radiation or dimethylbenzanthracene [34,35]. Although *Dmp1*-deficient mice develop a broad spectrum of epithelial and non-epithelial tumors, lung tumors were the most frequently encountered neoplasms in both *Dmp1*-null and *Dmp1*-hetrozygous mice [34,35]. The wild type *Dmp1* allele was retained and expressed in tumors arising from *Dmp1^+/-^* mice, demonstrating a haplo-insufficiency of Dmp1 in tumor suppression [35,51]. Tumors from *Dmp1*^-/-^ or *Dmp1^+/-^* (*Eμ-Myc*, *K-Ras^LA^*, *HER2* mutant) mice rarely showed mutations, deletions, or silencing of *p19^ARF^* or *p53*, suggesting that Dmp1 is a critical regulator of the *ARF*-p53 tumor suppressor pathway in vivo [14,34,35,39,48,49,50,51].

Activation of the *Dmp1* promoter by oncogenic Ras or mutant HER2 have been reported [11,14,36], indicating that it is a critical mediator in RAS or HER2 induced *ARF*, p53 cell cycle arrest to prevent incipient cancer cells. The *Dmp1* promoter was also activated by an inflammatory cytokine TNFα mediated by NF-κB [38] as well as dsDNA breaks [41], indicating that Dmp1 is a mediator of a variety of stress signaling. We conducted GeneChip microarray using *Dmp1^+/+^* and *Dmp1^-/-^* lungs and found that other transcriptional targets for Dmp1α include *Areg*, *Thbs1*, *JunB*, and *Egr1* [40], suggesting that it is involved in signal transduction pathways involving cell proliferation, angiogenesis, and invasion/metastasis.

Dmp1 shows it tumor suppressive activity not only transactivating the *ARF* promoter in response to oncoprotein overexpression, but also through physical interaction with p53 in response to DNA damage response [41]. Our data indicate that acceleration of DNA-binding of p53 by Dmp1 is a critical process for Dmp1 to increase the p53 function in *ARF*-deficient cells [47].

Whether h*DMP1* is involved in the pathogenesis in human cancer is a critical issue for research. We found that loss of heterozygosity (LOH) of h*DMP1* was present in ~40% of non-small cell lung carcinomas (NSCLC), especially those that retain wild type *INK4a/ARF* and/or *p53* [39]. In this study, we received specimen from the University Minnesota VA Hospital (UM) to study the survival of LOH for h*DMP1*, *ARF/INK4a*, *p53*; the impacts for overexpression of cyclin D1 and YY1 have also been studied on NSCLC survival, both progression-free survival (PFS) and total survival (TS).

The goals of this study are to clarify prognostic impact form the h*DMP1* LOH, *ARF/INK4a* LOH, p53 LOH and immunohistochemistry (IHC), Cyclin D1 (IHC), and YY1 (genomic DNA amplification).

## 2. Results

### 2.1. Impacts of LOH for hDMP1, ARF/INK4a, and p53 on NSCLC Survival

Previous publication from WFU with samples of 51 NSCLC [39] showed that LOH of the h*DMP1* gene was found in 33.3% of samples with 5′ primer, 36.1% with 3′ primer (average 35%), but signs of biallelic involvement (promoter hypermethylation or complete loss) was extremely rare (the former 2.2% and 0% for the latter) in human lung cancer indicating that h*DMP1* was haploinsufficient for tumor suppression [35,51]. Now, we have studied different set of samples (*n* = 70) with survival data (PFS and TS) from the University of Minnesota VA hospital (UM) [48,49].

**Figure 1:**
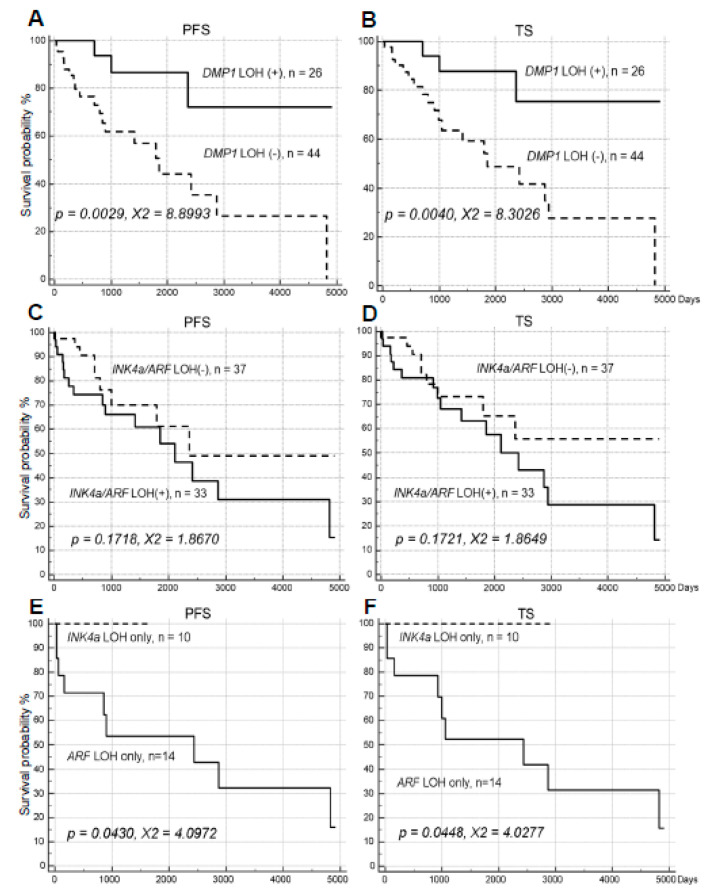
Progression-free Survival (PFS) and total survival (TS) of 70 cases of human non-small cell lung cancer obtained from the University of Minnesota VA hospital (UM) dependent on LOH for h*DMP1* (**A**,**B**) and *ARF/INK4a loci* (**C**–**F**). Kaplan-Meier analyses have been conducted to study the impact for the impact of loss of each locus on non-small cell lung cancer (NSCLC) patients’ disease-free survival up to 5000 days. The Med Calc software (Mariakerke, Belgium) was used to analyze the specimens. LOH for h*DMP1* (**A**,**B**) has significantly positive impact on patient’s relapse-free survival. On the other hand, LOH for *ARF/INK4a* did not have significant impact on NSCLC survival although there was a trend for association of worse prognosis (the survival indicate either of the two primers; **C**,**D**). When the *ARF/INK4a* locus was studied separately, the prognosis of patients with *ARF* LOH was much worse than that of *INK4a* only, indicating much stronger prognostic power for the former than the latter (**E**,**F**).

With the 5′ set of h*DMP1* primers (#92465), 10 of 70 cases (14.3%) were positive for LOH; with the 3′ set of h*DMP1* primers (#198004, #176671), 24 of 70 cases were positive (34.3%) (average 24.3%). 26 of 70 cases (37.1%) were positive for either of these (Table 1). The number was close to those published in WFU samples (35%; 39). Eight of 70 cases (11.4%) were positive for LOH with both of these h*DMP1* primer sets (Table 1), suggesting that gene deletion extends the entire *DMP1* locus in these samples. LOH for h*DMP1* was associated with longer survival in both PFS (*p* = 0.0029, χ^2^ = 8.8993) and total survival (*p* = 0.0040, χ^2^ = 8.3026) in the UM samples (Figure 1A,B). Consistently, LOH for h*DMP1* was associated with favorable survival in TS in our previous WFU samples analyzed (*p* = 0.0324, χ^2^ = 4.5230, *n* = 42, Supplementary Figure S1A) [39]. The tendency was more prominent is squamous cell carcinoma than adenocarcinoma (Supplementary Figure S2).

Then the same NSCLC samples pairs were studied for LOH of *ARF/INK4a* and *p53*. With *ARF/INK4a* primers, 23 of 70 cases (32.9%) were positive for LOH 5′ primers close to the *ARF* locus, 19 of 70 cases (27.1%) were positive for LOH 3′ primers close to the *INK4a* locus (average 30%), and 33 of 70 showed LOH for either one of these (47.1%). Nine of 70 cases (12.9%) showed LOH with both sets of the *ARF/INK4a* primers (Table 1). Importantly, LOH for the *ARF/INK4a* locus was not associated with survival PFS (*p* = 0.1718, χ^2^= 1.8670) or TFS (*p* = 0.1721, χ^2^= 1.8649) although there was a trend that it was associated with worse prognosis (Figure 1C,D). The trend was the same when AC and SCC were separately analyzed (Supplementary Figure S3). The LOH for the *ARF/INK4a* locus was not associated with TS in previous WFU specimens analyzed (*p* = 0.7707, χ^2^ = 0.08495, *n* = 43; Supplementary Figure S1B). Forty-seven of 53 cases (88.7%) showed mutually exclusive loss of the h*DMP1* and the *ARF-INK4a* loci (*p* = 0.0023; χ^2^ = 9.330; 95% confidence interval, 80.1%–97.2%; Table 1). We found that LOH for *ARF* was much worse prognostic factor than that of *INK4a* (Figure 1E,F) in both PFS and TS; as a matter of fact none of the NSCLC relapsed and died within 1800 (PFS) and 3000 days (TS) of observation for *INK4a* LOH, whereas NSCLC with *ARF* deletion relapsed and died in our survival analyses (Figure 1E,F; *p =* 0.0430 for PFS and *p =* 0.0448 for TS), suggesting that *ARF* LOH has much stronger impact on NSCLC survival than that of *INK4a*. When the survival for LOH for both of *ARF* and *INK4a* was studied, it was shorter in patients with the locus involvement than those without although data were not statistically significant (Supplementary Figure S4).

LOH of *p53* (incl. biallelic gene deletion) was found in 37.1% (26/70) with 5′ primers, 47.1% (33/70) with 3′ primers (average 42.1%), and 61.4% (43/70) with either primers in UM NSCLC samples (Table 1). LOH of *p53* was associated with worse prognosis in both PFS (*p* = 0.0472, χ^2^ = 3.9372) and TS (*p* = 0.0302, χ^2^ = 4.6963) in the UM samples (Supplementary Figure S5A,B). Consistently, LOH for *p53* had the trend for worse prognosis in WFU NSCLC samples (*p* = 0.1079, χ^2^ = 2.5849, *n* = 42; Supplementary Figure S1C). Forty-eight of 58 cases (82.8%) showed mutually exclusive loss of the h*DMP1* and the *p53* loci (*p* = 0.0128; χ^2^ = 6.195; 95% confidence interval, 75.6–93.9%; Table 1). The *p* values did not become smaller when AC and SCC were analyzed separately since N became smaller (Supplementary Figure S6).

We also conducted IHC analysis for p53 to study the impact of p53 expression on patients’ survival (Supplementary Figure S5C,D). High expression of p53 protein (level 2, intense staining) was associated with shorter survival in PFS (*p* = 0.0340, χ^2^ = 4.4944, *n* = 62) and TS (*p* = 0.0537, χ^2^ = 3.7270) suggesting that it is an indicator worse prognosis (Supplementary Figure S5C,D). The *p* vales were relative large because there were significant number of samples where p53 IHC was not performed, and because there were samples that showed weak p53 IHC (grade 1; these cases were excluded from the study). Like the relation of LOH for h*DMP1* and *p53* loci, LOH of h*DMP1* and overexpression of the p53 protein did not to overlap each other (29/33 = 87.9% mutually exclusive; 95% confidence interval: 76.7%–99.0%; Table 1).

### 2.2. The Impact of hDMP1 LOH on NSCLC Survival with p53 LOH

**Figure 2:**
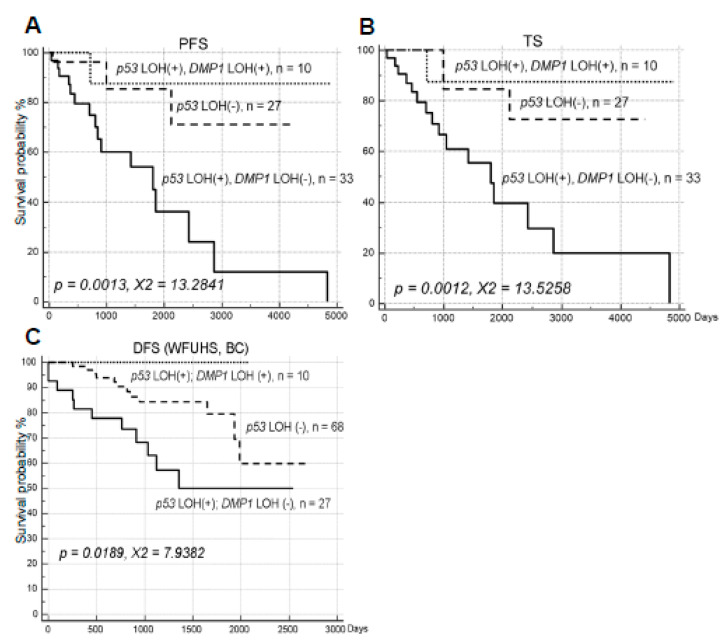
The triple analysis of cancer survival for p53 in University of Minnesota (UM) (NSCLC), and Wake Forest University (WFU) (breast cancer) samples. The impact of *DMP1* LOH on *p53* LOH was analyzed by Medcalc software for UM (NSCLC) and WFU (breast cancer) samples. Loss of *DMP1* neutralized the negative effect of *p53* LOH in NSCLC by moving the survival curves from *p53* LOH (+) to *p53* LOH(-) levels in both PFS and TS in UM samples. The same trend was observed in statistically significant fashion in WFU NSCLC samples (*n* = 44) or WFU breast cancer samples (*n* = 105) suggesting that the improvement of cancer survival of *p53* LOH samples with loss of the *DMP1* locus is a generalized phenomenon. (**A**) PFS of lung cancer (UM); (**B**) TS of lung cancer (UM), (**C**) DFS of breast cancer (BC, WFUHS).

The fact that LOH for h*DMP1* was mutually exclusive for that of p53 in 82.8% in NSCLC samples (48/58, Table 1) means that LOH for h*DMP1* was overlapping with that of *p53* in 17.2% of cases (10/58, 12 cases showed no LOH for *DMP1* and *p53*). We, therefore, studied the impact of h*DMP1* LOH in *p53* LOH(+) UM samples (Figure 2). We found that h*DMP1* LOH dramatically improved the survival (both PFS and TS) of *p53* LOH(+) in NSCLC patients’ survival in NSCLC (Figure 2A,B). As a matter of fact, the survival of *p53* LOH(+); h*DMP1* LOH(+) was comparable to or even better than that of *p53* LOH(-) in UM samples (*p* = 0.0013, χ^2^ = 13.2841 in PFS; *p* = 0.0012, χ^2^ = 13.5258 in TS, triple survival assay), indicating that one locus h*DMP1* deletion neutralized the negative effect of *p53* LOH in NSCLC (3000 day survival of 12.0% became 87.5% in PFS (7.3 fold improvement), Figure 2A; 3000 day survival of 19.8% became 87.5% in TS (4.2 fold), Figure 2B). The same phenomenon was also observed in previous WFU NSCLC specimens as analyzed for the total survival (Supplementary Figure S7A, *p* = 0.0714, χ^2^ = 5.2800, *n* = 42). Indeed, no SCLC patient with double LOH for *p53* and h*DMP1* relapsed in the observation period of 1900 days. The *p* value was relatively large because two Wake Forest University Health Sciences (WFUHS) NSCLC samples with double h*DMP1*; *p53* LOH (1990-10, 2005-308 in ref. [39]) did not have any survival data. The same trend was also found in breast cancer samples in WFUHS [42] since none of the 10 patients that showed dual LOH for *p53* and h*DMP1* relapsed during the 2100 days of observation period (*p* = 0.0189, χ^2^ = 7.9382, *n* = 105) (Figure 2C). Conversely, the LOH for the *ARF/INK4a* locus did not improve the negative effect of *p53* LOH in NSCLC survival (Supplementary Figure S7B,C). These results show that the adverse prognostic impact of *p53* LOH in epithelial tumors (i.e., NSCLC and breast carcinoma) is greatly improved with simultaneous loss of the h*DMP1*, the result of which are consistent with favorable impact of h*DMP1* LOH in NSCLC (this study) and breast cancer [42].

### 2.3. Cyclin D1 Overexpression and YY1 Amplification and NSCLC Survival

**Figure 3:**
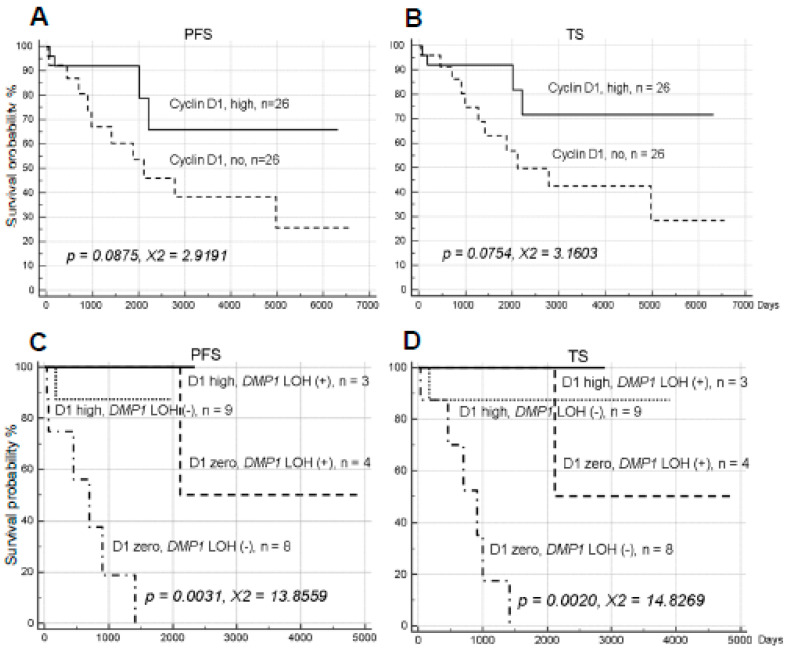
Progression-free survival (PFS) and total survival (TS) analysis of human NSCLC obtained from UM dependent on Cyclin D1. (**A**,**B**) PFS and TS analysis of human NSCLC obtained from UM dependent on Cyclin D1 in human NSCLC (*n* = 62). High expression of Cyclin D1 protein tended to be associated with longer survival. (**C**,**D**) The quadruple analysis of NSCLC samples on Cyclin D1 expression and LOH for *DMP1*. Quadruple analysis was conducted in UM NSCLC samples for Cyclin D1 high (2+); *DMP1* LOH (+) and (-), and Cyclin D1 no expression; *DMP1* LOH (+) and (-). Both Cyclin D1 expression and LOH for *DMP1* are associated with favorable prognosis; we could identify NSCLC patients with worst prognosis in the group of Cyclin D1, no expression; *DMP1* LOH (-) group.

Finally, we examined the impact of Cyclin D1 [52,53] overexpression at protein levels and *YY1* [54,55] expression at genomic DNA level. Overexpression of the Cyclin D1 protein (grade 2 in IHC) was found in 26 of 52 cases while 26 samples had no expression of the protein in NSCLC samples. Samples with Cyclin D1 overexpression had the trend to be associated with favorable prognosis in PFS and TS, but the data were not statistically significant (*p* = 0.0875, χ^2^ = 2.9191 in PFS; *p* = 0.0754, χ^2^ = 3.1603 in TS) (Figure 3A,B). Although both h*DMP1* LOH and Cyclin D1 overexpression were associated with favorable prognosis, the Cyclin D1 protein overexpression was not related to the LOH of h*DMP1* (*p* > 0.20) suggesting that these two events happened independently. When we conducted quadruple analysis of NSCLC for Cyclin D1 and h*DMP1* LOH, we found significantly shorter survival for the group without Cyclin D1 expression without LOH for h*DMP1* (*p* = 0.0031, χ^2^ = 13.8559 for PFS; *p* = 0.0020, χ^2^ = 14.8269 for TS; Figure 3C,D). As a matter of fact, none of the patients lived more than 1400 days in this group where clinicians are alerted for early relapse.

**Figure 4:**
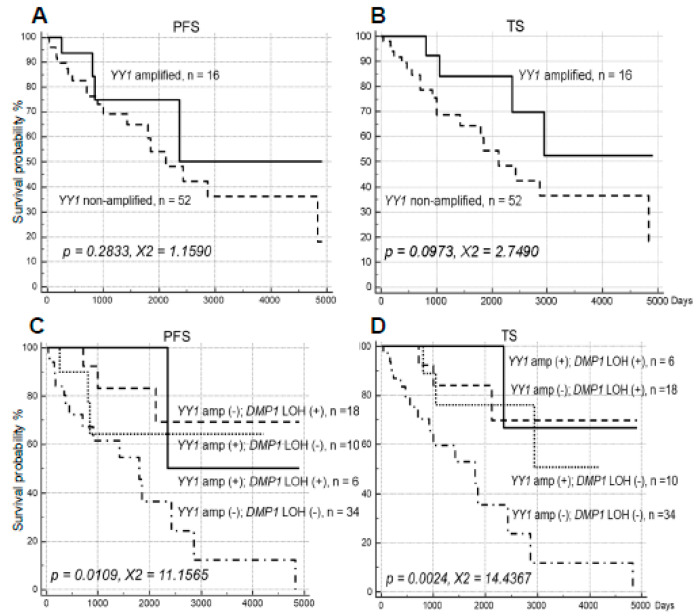
PFS and TS analysis of human NSCLC obtained from UM dependent on *YY1*. (**A**,**B**) PFS and TS analysis of human NSCLC obtained from UM dependent on *YY1* in human NSCLC. (**C**,**D**) The quadruple analysis of NSCLC samples on *YY1* amplification and LOH for *DMP1*. Quadruple analysis was conducted in UM NSCLC samples for *YY1* amplification (>3 folds); *DMP1* LOH (+) and (-); *YY1* no-amplification; *DMP1* LOH (+) and (-); *YY1* amplification. Both *YY1* amplification and LOH for *DMP1* are associated with favorable prognosis; we could identify NSCLC patients with worst prognosis in the group of *YY1* no-amplification; *DMP1* LOH (-) group.

The MDM2 stimulator [54] and epigenetic modifier [55]. *YY1* gene amplification (>3 folds) was found in 16 of 68 NSCLC samples (23.5%) examined. The gene amplification seemed to predict favorable outcome of patients, but neither of them (PFS: *p* = 0.2833, χ^2^ = 1.1590; TS: *p* = 0.0973, χ^2^ = 2.7490) was statistically significant (Figure 4A,B). Amplification of *YY1* genomic locus was independent of LOH for h*DMP1* (*p* > 0.50). Then we conducted quadruple analysis of NSCLC for *YY1* amplification and h*DMP1* LOH. We found shorter survival for the group without *YY1* amplification expression without LOH for h*DMP1* (*p* = 0.0109, χ^2^ = 11.1565 for PFS, *p* = 0.0024, χ^2^ = 14.4367 for TS; Figure 4C,D), indicating that *YY1* amplification (-); h*DMP1* LOH (-) genotype is an ominous sign of NSCLC.

In summary, both Cyclin D1 protein overexpression and *YY1* genomic amplification seem to predict favorable outcome of NSCLC patients; these do not overlap with LOH for h*DMP1* which is another favorable prognostic factor. When these are absent without LOH for h*DMP1*, NSCLC patients will not live long.

## 3. Discussion

We have analyzed LOH values for h*DMP1, ARF/INK4a,* and *p53 loci* from NSCLC specimen obtained from a different institution. The percentage is a bit different—37.1% of h*DMP1* locus, 47.1% for *ARF/INK4a*, and 61.4% for *p53* in the UM VA hospital samples collected in 1991–2000 while it was 40.8%, 36.0%, and 47.9% from the WFU hospital, 1999–2006, because of the difference of demographic distribution. Importantly, the h*DMP1* locus had positive impact on NSCLC survival in both institutions (*p* = 0.0029 in PFS, *p* = 0.0040 in TS in UM samples; *p* = 0.03551 in WFU sample) [39]. LOH for the *ARF/INK4a* locus did not have prognostic values in both institutions (*p* = 0.1718 in PFS, *p* = 0.1721 in TS in UM specimens), although there was a trend that LOH for *ARF/INK4a* locus with either of the two primers was associated with worse prognosis in UM samples. Current data show that LOH for the *ARF/INK4a* locus is mutually exclusive for that for the h*DMP1* (88.7%) in NSCLC. Published studies show that Dmp1 activates both *ARF*-p53 and Ink4a-Rb pathways in mice for tumor suppression [43]. These human and mouse data are very consistent indicating that DMP1 is in the upstream of these pathways, both having binding sites for transactivation [43].

Since *ARF/INK4a* locus 5′ probe #33647 was close to *ARF* exon 1β and #27251 was close to *INK4a* exon 1α, LOH was evaluated separately in this study. Of note, we noticed that #33647 LOH (representing the *ARF* locus; LOH, 23/70, 32.9%) had much worse impact on NSCLC survival than that of #27251 (representing the *INK4a* locus; 19/70, LOH 27.1%). This is quite unexpected because INK4a has been considered to be much more important tumor suppressor than *ARF* in human cancer [21,22]. This is possibly because the *ARF* gene is inactivated in human cancer by gene deletion, splicing alteration than *INK4a,* which is inactivated mainly by promoter methylation or coding exon point mutations [5,16]; for splicing errors for *ARF*, see ref. [2]). Frameshift caused by nucleotide insertion or deletion affects both of these genes at equal frequency [16]. Since the frequency of LOH is similar for *ARF* and *INK4a*, it is highly possible that *ARF* plays an important role as *INK4a* in suppressing tumor development in NSCLC. Importantly, LOH for *ARF/INK4a* was mutually exclusive of that for the h*DMP1* locus in UM samples (this study) as well as WFU samples [39,42] confirming that DMP1 is in the same pathway as that of *ARF*-p53 signaling.

In good contrast to LOH for h*DMP1*, LOH for *p53* had negative impact on patients’ survival (Supplementary Figure S5) in both PFS and TS in UM NSCLC samples. Likewise, IHC study shows that p53 protein overexpression is associated with worse prognosis in lung cancer. Again IHC staining for p53 was mutually exclusive for that of h*DMP1* in UM specimen for NSCLC (29/33). The data are consistent our findings that Dmp1 binds directly to the p53 protein, esp. when cells receive DNA damage [41,47]. IHC for p53 (grade 2) tend not to overlap LOH for h*DMP1* in our study (87.9% exclusive), indicating that p53 overexpression is a sign of p53 mutation(s) that happen(s) with LOH for *p53*.

We also conducted survival analysis of h*DMP1*; *p53* double LOH cases (*n* = 10 in UM samples) although the analysis was difficult due to mutual exclusiveness of LOH for h*DMP1* and *p53*. We expected that when inactivation of two tumor suppressors overlap, the prognosis of patients will be even worse than *p53* LOH alone; however, the negative effect of *p53* loss was strikingly improved by simultaneous loss of h*DMP1* (Figure 2). The trend was the same regardless of the origin of NSCLC samples (UM or WFU) or the cancer types (lung cancer or breast cancer). The improvement of the negative effects of *p53* LOH in NSCLC was specific to h*DMP1* LOH because it was not found in LOH for *ARF/INK4a* (Supplementary Figure S7B,C). The quenching of poor prognosis of *p53* LOH by simultaneous loss of h*DMP1* is consistent with relatively good prognosis of NSCLC with LOH for h*DMP1* in both institutions. Although the molecular mechanism(s) underlying this phenomenon is not clear at this moment, we speculate that it is a generalized tendency of carcinoma. Our data show that Dmp1α stimulates the p53 pathway through direct physical interaction with p53 in response to dsDNA breaks [41,47]. The human *DMP1* locus encodes *DMP1α*, and its splice variants *DMP1β* and *γ* [46]. Our preliminary study suggests that Dmp1α binds to both wt and mutant p53 although the affinity is much higher for the latter. Thus, DMP1α is either tumor suppressive (wt p53) or oncogeic (mutant p53) dependent on the p53 status of cells. DMP1β/γ do not interact with p53, is always oncogenic [45]. DMP1α regulates the human *ARF* promoter, the activity of which is antagonized its splice variant DMP1β [46]. Whatever the mechanism is, small molecule inhibitor screening should be performed to disrupt DMP1α and mutant p53 interaction for future cancer therapy.

The present study shows that Cyclin D1 overexpression as detected by IHC is associated with longer survival/better prognosis of NSCLC in both PFS and TS. The *p* values were more than 0.05 because we cannot include low level of Cyclin D1 expression (grade 1) and because not all samples were stained for Cyclin D1 in the current study. The current study shows that both h*DMP1* LOH and Cyclin D1 overexpression are favorable prognostic factors associated with longer survival (Figure 1 and Figure 4). Thus we conducted quadruple analysis of NSCLC patients and found that samples that show Cyclin D1 IHC zero and h*DMP1* LOH (-) have much worse prognosis than other three groups (Figure 4C,D). It is expected that tumor cells without LOH for h*DMP1* and Cyclin D1, low express high hDMP1. Whatever the situation is, hDMP1 overexpression combined with no Cyclin D1 expression in IHC is associated with worse prognosis of NSCLC as shown in Figure 3C,D, delineating special group of NSCLC patients who will relapse early. Dmp1α behaves as a tumor suppressor when the p53 is wild type since it binds and stabilizes p53, but it will behave like as an oncogene when p53 is mutant. Whatever the situation is, molecular studies should be performed in the near future to explain the oncogenic role of hDMP1 in human cancer. Of note, mouse genome produces only Dmp1α, but not β or γ isoforms, explaining the difference between mice and humans.

We also studied the effects of *YY1* genomic amplification in NSCLC. Its amplification again had positive effects in NSCLC survival although the data were not statistically significant. It has been reported that YY1 augments HDM2-mediated p53 polyubiquitination, and thus be oncogenic [54]. Our study shows that Dmp1α antagonizes p53’s ubiquitination by MDM2 both in vitro and in cell, and restores p53’s nuclear localization that had been lost with MDM2 expression [41]; theoretically Dmp1α will antagonize the action of YY1 to show its tumor-suppressive activity. Then the LOH for h*DMP1* will nullify the effects of YY1 in NSCLC survival. As a matter of fact, the survival of *YY1*-amplified lung cancer almost the same between *YY1* amplified cases and *YY1* amplified; h*DMP1* LOH cases in PFS and TS of NSCLC. We identified a group of bad prognosis in NSCLC, i.e., NSCLC without *YY1* amplification without LOH for h*DMP1* had the worse prognosis than the other three groups.

We will analyze mRNA levels in the future.

## 4. Materials and Methods

### 4.1. Human Lung Cancer Samples

Seventy pairs of frozen human lung cancer tissues (36 cases of adenocarcinoma, 25 cases of squamous cell carcinoma, 5 cases of large cell carcinoma, and 4 cases of adenosquamous carcinoma) and their normal counterparts were obtained from the Tissue Procurement Core Facility at the Minnesota Veteran’s hospital (UM VA hospital [56,57]). Fifty-one pairs of frozen human lung cancer tissues (33 adenocarcinoma, 16 squamous cell carcinoma, and 2 adenosquamous carcinoma) and their normal counterparts were obtained from the Tissue Procurement Core Facility at the Wake Forest University Comprehensive Cancer Center (WFU [39]). The samples had already been resected from patients with informed consent and had been stored in liquid nitrogen in both cases. The samples do not contain any subject identifiers. The human protocol had been approved by the Institutional Review Board.

### 4.2. Loss of Heterozygosity (LOH) and Sequencing Analyses of Human Lung Cancer Specimen

LOH assays have been conducted as described previously by PCR [39,42]. PCR products were visualized on a 1.2% agarose gel. Genotypes were identified by peak analysis of the fluorescent signal detected on an ABI 3700 DNA analyzer (Applied Biosystems). LOH was assessed if the qLOH value was found to be >2.0 or <0.5 [39,42]. The third set of primers (#176671; Table 1) was used to examine the status of hDMP1 gene 3′ when LOH study of h*DMP1* locus was single with those for #198004. Real-time PCR was also conducted to study the genomic status (shown as deletion, no deletion in the Table 1).

### 4.3. Statistical Analyses for Mutual Exclusiveness of LOH

Chi square analyses and confidence interval assays were performed as previously described [39]. IHC studies were also performed for Cyclin D1 [43,52,53] and gene amplification study was performed for exon 4 of human *YY1* [54,55]. The cut-off level for *YY1* was 3.0 folds over neighbor tissues in survival analyses.

The progression-free survival (PFS) and total survival (TS) of UM NSCLC specimen was analyzed by using MedCalc software (Ostend, Belgium; 42). The TS of WFU specimens were also analyzed by the MedCalc software.

## 5. Conclusions

We have done survival analyses of NSCLC patients in UM and WFU samples, the data of which were obtained by LOH and IHC analyses. LOH for h*DMP1* was associated with favorable prognosis while that of *p53* with worse prognosis. LOH for *ARF* had much negative effects than *INK4a* loss in NSCLC survival, showing the role of *ARF* tumor suppressor by gene deletion. Our data also show that the adverse effect of *p53* LOH was neutralized by simultaneous loss of the h*DMP1* locus. We are currently unaware of the mechanism(s), which will allow more bench works employing DMP1 and p53.

## Figures and Tables

**Table 1:** NSCLC samples of UM VA hospital. The LOH values of each genomic locus is shown. When both loci shows a single peak, real-time PCR was conducted to determine gene deletion. NME: Non-mutually exclusive, ME: Mutually exclusive. IHC: Immunohistochemistry. Not only chi square tests, but also 95% confidence interval assays were conducted to evaluate mutual exclusiveness of LOH for genomic loci. Mutually exclusive cases for LOH and immunohistochemistry (IHC), the results of confidence interval assays are shaded.

Patient ID	h*DMP1*		*INK4a/ARF*	*INK4a/ARF*	*I/A* LOH ME	*p53*		*p53* LOH ME	p53 IHC	p53 IHC ME
	**#92465**	**#198004**	**#33647**	**#27251**		**#182 SE**	**#377 SE**			
**Group 1**	5′	3′	5′	3′		5′	3′			
307	0.68	**0.26**	**0.15**	0.98	NME	**2.01**	**2.04**	NME	ND	
308	0.70	1.04	**3.38**	**0.41**	ME	**Deletion**	**27.20**	ME	ND	
309	1.21	**2.51**	1.42	1.11	ME	1.06	0.54	ME	ND	
310	**0.06**	**4.31**	0.68	0.93	ME	1.06	0.92	ME	ND	
311	1.05	1.08	1.31	1.26	None	1.50	0.84	None	ND	
313	0.94	**0.47**	1.46	1.32	ME	1.23	0.96	ME	ND	
314	**0.46**	**6.09**	**2.41**	0.63	NME	**0.43**	**0.30**	NME	ND	
315	1.09	1.87	1.12	0.98	None	**9.97**	**0.28**	ME	ND	
317	0.61	**2.26**	1.56	0.91	ME	**Deletion**	1.40	NME	ND	
318	1.23	0.90	**5.48**	**2.21**	ME	**0.42**	**0.38**	ME	ND	
319	1.86	**2.29**	1.30	0.82	ME	**2.16**	**3.57**	NME	ND	
321	0.77	**0.29**	0.69	1.03	ME	1.26	1.74	None	ND	
322	1.12	**2.01**	0.97	0.93	ME	1.16	**0.07**	NME	ND	
323	0.86	0.52	0.75	1.06	None	1.08	1.56	None	ND	
324	0.64	**2.96**	0.77	**0.16**	NME	**2.31**	**0.31**	NME	ND	
325	1.10	1.51	**2.13**	**0.45**	ME	**0.34**	**0.29**	ME	ND	
326	1.00	0.59	**0.28**	0.60	ME	**2.37**	0.62	ME	ND	
327	1.32	**>10.0**	1.04	1.03	ME	1.80	No deletion	ME	ND	
328	**2.33**	**4.97**	1.53	1.34	ME	No deletion	No deletion	ME	2	NME
329	0.74	0.80	1.02	**2.69**	ME	**0.25**	0.9	ME	2	ME
330	0.87	0.72	0.96	0.64	None	0.88	**13.97**	ME	1	None
331	1.11	1.08	**0.10**	**4.66**	ME	1.34	**0.48**	ME	0	None
332	0.82	0.52	0.68	0.74	None	0.97	**0.40**	ME	2	ME
333	1.15	**0.48**	0.89	1.06	ME	1.89	1.09	ME	1	ME
277	**0.41**	**0.41**	1.19	1.02	ME	0.61	No deletion	ME	1	ME
295	0.74	**0.13**	1.24	1.01	ME	0.99	1.02	ME	1	ME
299	**0.32**	**2.11**	1.18	0.79	ME	0.87	0.51	ME	1	ME
338	1.73	1.27	1.54	**0.04**	ME	**13.1**	**4.06**	ME	0	None
349	**2.95**	**20.00**	0.93	1.21	ME	1.73	1.62	ME	1	ME
351	1.69	**0.41**	No deletion	No deletion	ME	0.83	1.08	ME	1	ME
379	0.98	**0.18**	0.76	0.64	ME	0.92	0.94	ME	2	NME
380	**0.33**	**0.33**	0.67	0.80	ME	0.55	1.82	ME	ND	
271	0.66	0.59	**0.49**	0.91	ME	0.65	**3.39**	ME	2	ME
272	0.87	0.92	0.78	1.93	None	0.92	0.82	None	0	None
273	0.57	**0.41**	**0.32**	0.83	NME	**0.23**	**Deletion**	NME	2	NME
274	1.02	0.87	0.70	0.95	None	0.69	**0.17**	ME	1	None
275	0.55	0.90	**0.18**	1.04	ME	**0.35**	**Deletion**	ME	2	ME
278	0.95	No deletion	0.76	**3.11**	ME	0.56	**14.00**	ME	2	ME
282	1.12	1.02	1.03	0.95	None	0.93	**4.01**	ME	2	ME
284	1.02	1.02	**0.35**	0.92	ME	**2.27**	**10.40**	ME	0	None
290	1.03	0.92	**2.48**	1.03	ME	0.96	0.92	None	2	ME
293	0.94	0.99	0.60	0.73	None	1.86	**0.47**	ME	0	None
**Group 2**										
269	0.74	0.74	0.76	**0.15**	ME	0.71	**0.33**	ME	2	ME
270	1.08	1.16	1.10	0.99	None	0.73	1.06	None	2	ME
276	0.85	1.07	**4.00**	1.41	ME	**0.15**	**3.08**	ME	2	ME
300	1.49	0.96	1.30	0.66	None	**2.23**	0.57	ME	2	ME
334	0.80	0.61	**0.35**	0.94	ME	1.05	**4.73**	ME	2	ME
337	1.06	0.81	1.09	0.88	None	1.66	**2.07**	ME	2	ME
339	0.66	1.75	1.27	**0.07**	ME	0.93	0.95	None	2	ME
340	**3.90**	0.91	1.30	1.25	ME	1.71	No deletion	ME	1	ME
353	**4.28**	**0.37**	1.20	0.59	ME	**3.62**	No deletion	NME	1	ME
356	1.68	1.03	1.65	0.59	None	No deletion	No deletion	None	0	None
357	1.17	1.26	1.88	**0.18**	ME	**2.59**	**2.55**	ME	1	None
358	1.07	1.23	**2.91**	1.45	ME	**2.01**	0.65	ME	1	None
360	0.95	1.02	**0.25**	**0.41**	ME	**0.45**	**3.11**	ME	2	ME
362	1.64	No deletion	1.33	1.03	None	0.88	**0.22**	ME	2	ME
363	0.84	0.88	**0.27**	1.22	ME	**0.35**	**0.18**	ME	1	None
364	1.04	1.07	**0.11**	**0.06**	ME	0.75	**0.16**	ME	0	None
365	No deletion	0.93	1.29	0.95	None	0.75	0.93	None	1	None
366	0.87	1.26	**0.44**	0.99	ME	1.04	0.98	None	2	ME
367	0.92	1.04	0.78	**2.15**	ME	0.70	1.20	None	0	None
368	0.97	**0.48**	**0.35**	**0.27**	NME	1.52	0.91	ME	1	ME
369	1.49	1.06	1.40	1.27	None	**3.42**	No deletion	ME	0	None
370	0.55	1.11	1.76	**0.26**	ME	**0.47**	No deletion	ME	2	ME
371	1.49	0.56	**8.11**	**2.46**	ME	0.71	**0.25**	ME	0	None
372	**2.50**	1.72	0.51	0.57	ME	**0.27**	No deletion	NME	2	NME
381	1.30	0.99	1.02	1.19	None	0.55	**0.49**	ME	0	None
384	1.63	0.80	**2.53**	**0.48**	ME	1.54	1.38	None	2	ME
387	1.23	**0.26**	**0.39**	1.99	NME	**2.04**	1.80	NME	0	ME
389	1.14	1.23	1.47	**0.17**	ME	1.16	**0.42**	ME	1	None
Percentage		26/70 = 37.1%		33/70 = 47.1%	47/53 = 88.7%		43/70 = 61.4%	48/58 = 82.8%		29/33 = 87.9%
					*p = 0.0023*			*p = 0.0128*		
					X2 = 9.330			X2 = 6.195		
				95% CI	80.1–97.2%		95% CI	75.6–93.9%		76.7–99.0%

## Supplementary Materials

Supplementary Materials can be found at https://www.mdpi.com/1422-0067/21/21/7971/s1. Supplementary Figure S1. Total survival (TS) of patients of human non-small cell lung cancer obtained from the Wake Forest Baptist hospital dependent on LOH for h*DMP1* (A), *ARF*/*INK4a* (B), and *p53 loci* (C). Kaplan-Meier analyses have been conducted to study the impact for of loss of each locus on non-small cell lung cancer (NSCLC) patients’ total survival up to 3500 days. The MedCalc software (Mariakerke, Belgium) was used to analyze the specimens. LOH for h*DMP1* (A, *n* = 42) has significantly positive impact on patient’s relapse-free survival. On the other hand, LOH for *ARF*/*INK4a* did not have significant impact on NSCLC survival (the TS indicate either of the two primers; B, *n* = 43). *p53* LOH had statistically significant negative impact on NSCLC patients’ survival (C, *n* = 42); Supplementary Figure S2. PFS and TS of patients of human AC and SCC of the lung cancer obtained from the Minnesota VA Hospital on LOH for h*DMP1*. The quadruple analyses; Supplementary Figure S3. PFS and TS of patients of human AC and SCC of the lung cancer obtained from the Minnesota VA Hospital on LOH for *ARF*/*INK4a* (either). The quadruple analyses; Supplementary Figure S4. PFS and TS of patients of human non-small cell lung cancer obtained from the Minnesota VA Hospital on LOH for *ARF*/*INK4a* (both). PFS and TS were determined by the MedCalc software. Two groups comparison was made between that of *ARF*/*INK4a* LOH (-) and *ARF*/*INK4a* LOH, both (+) (A, PFS; B, TS); Supplementary Figure S5. PFS and TS of human NSCLC obtained from UM dependent on LOH and IHC for *p53*. Kaplan-Meier analyses have been conducted to study the impact for the impact of loss of the *p53* locus on NSCLC patients’ disease-free survival up to 5000 days. The same software was used to calculate the p and chi-square values. *p53* LOH had statistically significant negative impact on NSCLC patients’ survival (A,B). Similar analyses were conducted with IHC data for the *p53* protein which often overlap with LOH for *p53* (C,D). The *p* values were relatively larger because not all samples were stained for *p53*, and because grade 1 staining was excluded from the study; Supplementary Figure S6. PFS and TS of patients of human AC and SCC of the lung cancer obtained from the Minnesota VA Hospital on LOH for *p53* (either). The quadruple analyses; Supplementary Figure S7. The triple analysis of cancer survival for *p53* in WFU (NSCLC, A), UM (NSCLC; B,C) samples. The impact of *DMP1* LOH on *p53* LOH was analyzed by Medcalc software for WFU and UM NSCLC samples. Loss of *DMP1* neutralized the negative effect of *p53* LOH in NSCLC by moving the survival curves from *p53* LOH (+) to *p53* LOH (-) levels or better in WFU samples. On the other hand, both PFS and TS of *p53* LOH became worse by simultaneous LOH for *ARF*/*INK4a* (B,C).
