# Supplementary material for: Survival of Lung Cancer Patients Dependent on the LOH Status for DMP1, ARF, and p53
Source: Int J Mol Sci. 2020 Oct 27;21(21):7971. doi: 10.3390/ijms21217971 (PMC7662351; doi:10.3390/ijms21217971)
Supplement: Supplementary file 1 [file ijms-21-07971-s001.pdf]

Supplementary Figure S1. Total survival (TS) of patients of human non-small cell lung cancer obtained from the Wake Forest Baptist hospital dependent on LOH for h*DMP1* (A), *ARF*/*INK4a* (B), and *p53* loci (C).

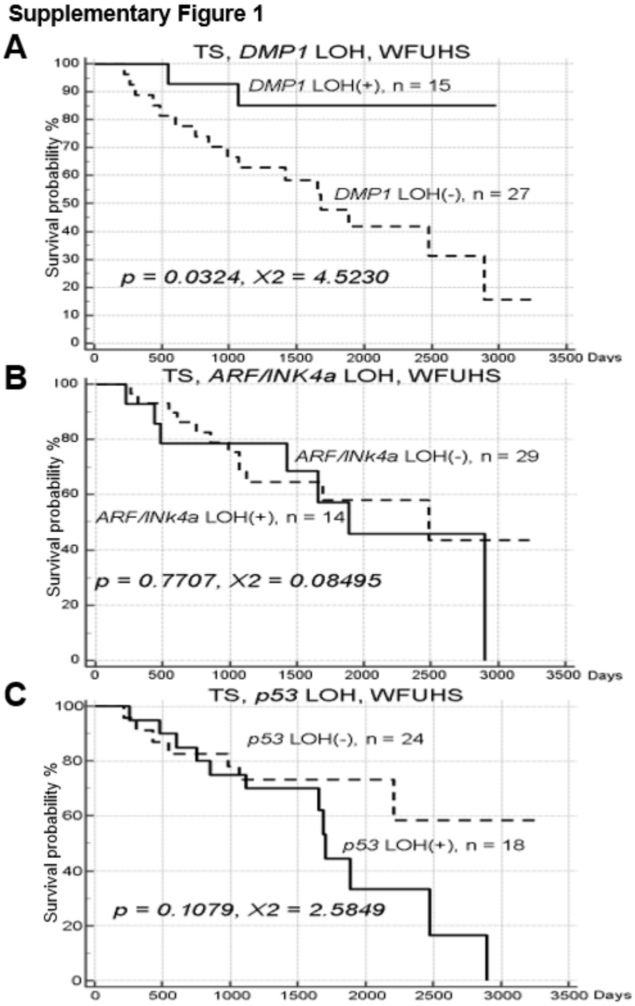

Kaplan-Meier analyses have been conducted to study the impact for of loss of each locus on non-small cell lung cancer (NSCLC) patients' total survival up to 3,500 days. The MedCalc software (Mariakerke, Belgium) was used to analyze the specimens. LOH for h*DMP1* (A, n = 42) has significantly positive impact on patient's relapse-free survival. On the other hand, LOH for *ARF*/*INK4a* did not have significant impact on NSCLC survival (the TS indicate either of the two primers; B, n = 43). *p53* LOH had statistically significant negative impact on NSCLC patients' survival (C, n = 42).

Supplementary Figure S2. PFS and TS of patients of human non-small cell lung cancer obtained from the Minnesota VA Hospital on LOH for *ARF*/*INK4a*.

Supplementary Figure 2

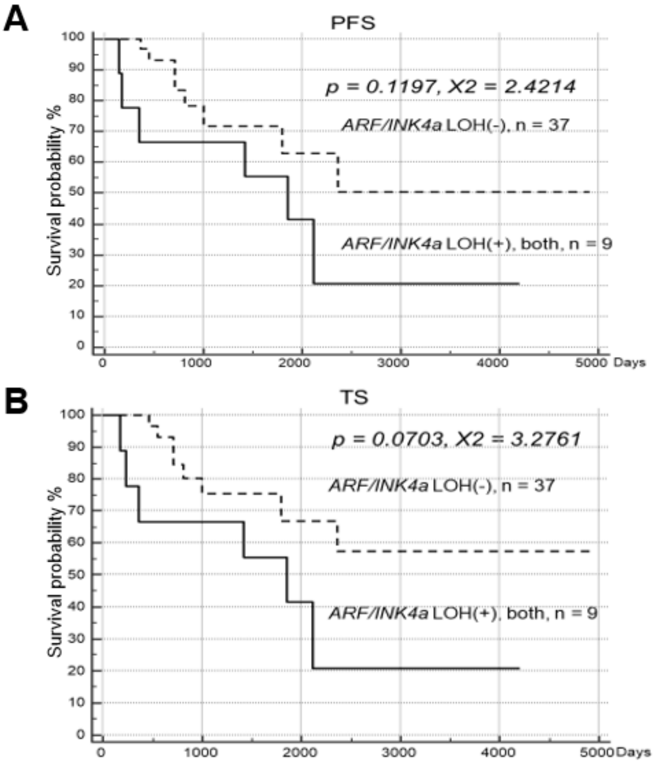

PFS and TS were determined by the Med Calc software. Two groups comparison was made between that of *ARF*/*INK4a* LOH(-) and *ARF*/*INK4a* LOH, both (+) (A, PFS; B, TS).

Supplementary Figure S3. PFS and TS of human NSCLC obtained from UM dependent on LOH and IHC for *p53*.

Supplementary Figure 3

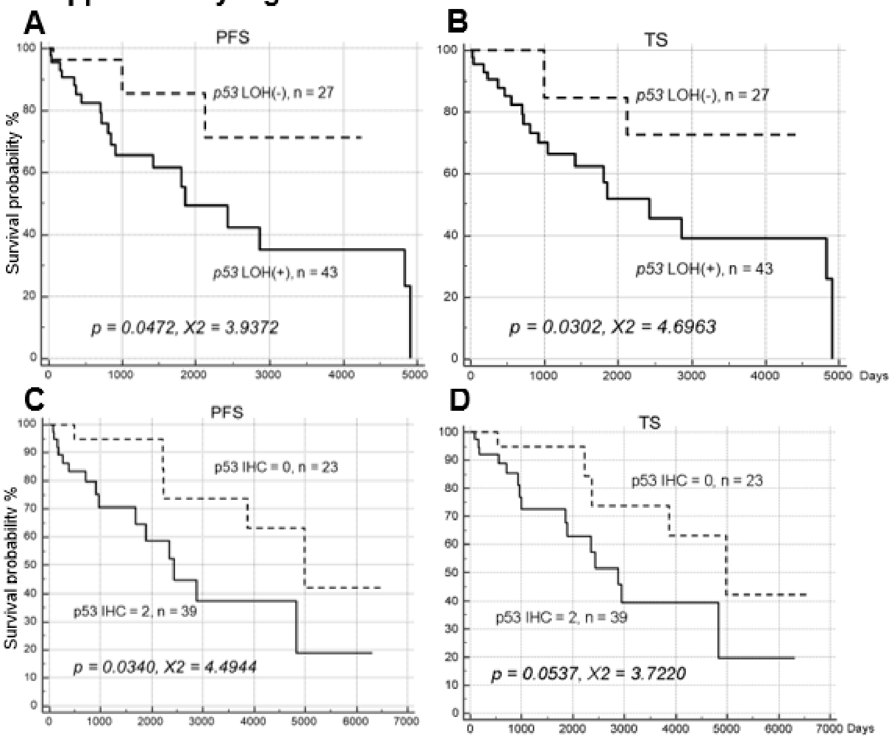

Kaplan-Meier analyses have been conducted to study the impact for the impact of loss of the *p53* locus on NSCLC patients' disease-free survival up to 5,000 days. The same software was used to calculate the *p* and chi-square values. *p53* LOH had statistically significant negative impact on NSCLC patients' survival (A, B). Similar analyses were conducted with IHC data for the p53 protein which often overlap with LOH for *p53* (C, D). The *p* values were relatively larger because not all samples were stained for p53, and because grade 1 staining was excluded from the study.

Supplementary Figure S4. The triple analysis of cancer survival for p53 in WFU (NSCLC, A), UM (NSCLC; B, C) samples.

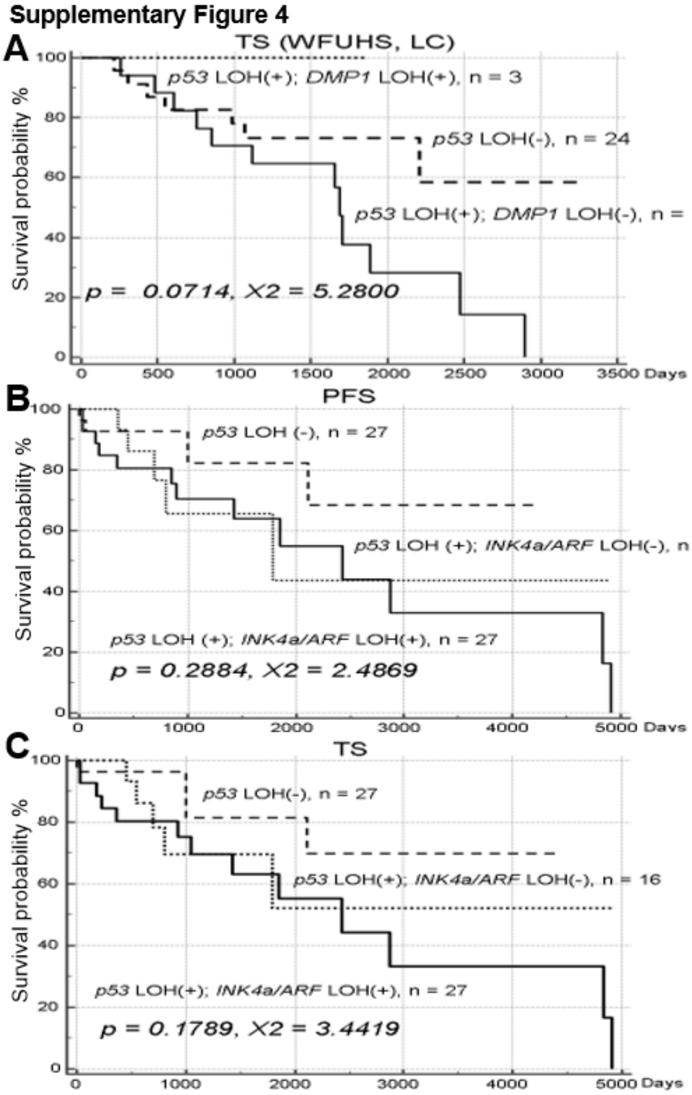

The impact of *DMP1* LOH on *p53* LOH was analyzed by Medcalc software for WFU and UM NSCLC samples. Loss of *DMP1* neutralized the negative effect of *p53* LOH in NSCLC by moving the survival curves from *p53* LOH(+) to *p53* LOH(-) levels or better in WFU samples. On the other hand, both PFS and TS of *p53* LOH became worse by simultaneous LOH for *ARF*/*INK4a* (B, C).
